# Reactions of a Dioxidomolybdenum(VI) Complex with Thionation Reagents—Formation of Mo(IV) Species with Sulfur Donors

**DOI:** 10.3390/molecules27217154

**Published:** 2022-10-22

**Authors:** Esko Salojärvi, Anssi Peuronen, Narhari Sapkota, Ari Lehtonen

**Affiliations:** Intelligent Materials Chemistry Research Group, Department of Chemistry, University of Turku, FI-20014 Turku, Finland

**Keywords:** molybdenum, sulfur donor, Lawesson’s reagent, transition metal complexes

## Abstract

Molecular molybdenum complexes with sulfur donor ligands are generally studied as soluble model compounds for molybdenum enzymes essential for life. The dioxidomolybdenum(VI) complex with tetradentate aminobisphenolate ligand undergoes a reaction with thionation reagent P_2_S_5_ or its organic derivative, Lawesson’s reagent, to yield stable Mo(IV) aminobisphenolate complexes, where pristine oxido ligands have been replaced by bidentate sulfur donors tetrasulfide, S_4_^2−^ or (4-methoxyphenyl)phosphonotrithioate residue derived from Lawesson’s reagent. This is in contrast to the behaviour of analogous dioxidotungsten(VI) complex, which, under similar conditions, yields W(VI) S_2_ systems. The overall *cis*,*trans*,*cis* geometry of the parent dioxidomolybdenum(VI) aminobisphenolate is retained, namely, the neutral nitrogen donors are in *cis* positions, phenolate oxygens are *trans* to each other and sulfur donors are *cis*. Although formally Mo(IV), thus d^2^ system, the studied complexes have diamagnetic singlet electron configurations as a result of the axially compressed octahedral structures.

## 1. Introduction

Molybdenum sulfides are useful catalysts in a number of important reactions used to activate small molecules, e.g., hydrogen evolution reaction, CO_2_ reduction and N_2_ fixation [1]. Moreover, molybdenum–sulfur bonds are found in all molybdenum enzymes; therefore, molecular molybdenum sulfide complexes are investigated as soluble model compounds for the active sites of such catalysts as well as for metal enzymes [2,3,4,5]. In principle, diverse metal–organic sulfide complexes can be synthesized by the reaction of simple metal sulfides and ligand precursors, but in practice, they are mostly made of easily available oxido complexes by simple thionation reactions, i.e., oxido-to-sulfido substitutions. Typically, the thionations are run using H_2_S [6,7,8,9,10], B_2_S_3_ [11,12,13,14], (R_3_Si)_2_S/R_3_SiSH [15,16,17,18], P_2_S_5_ [12], or Lawesson’s reagent (2,4-bis(4-methoxyphenyl)-1,3,2,4-dithiadiphosphetane-2,4-disulfide) [9,19]. We have earlier used the reaction of dioxidotungsten(VI) complexes with P_2_S_5_ and its soluble derivative, Lawesson’s reagent, to prepare stable disulfidotungsten(VI) complexes supported with tetradentate aminobisphenolates [20]. As equivalent Mo and W dioxido compounds have, in many cases, very similar molecular and crystals structures and rather similar chemical properties, we might expect parallel reactivity in the thionation reactions as well. However, under identical reaction conditions, the dioxidomolybdenum(VI) aminobisphenolates did not yield the anticipated disulfido derivatives; instead, the reactions led to the reduction of the metals’ centres as the isolated products were identified as Mo(IV) complexes. In this article, we report the reactivity of dioxidomolybdenum(VI) aminobisphenolate with thionation reagents and structural characterization of two Mo(IV) species with sulfur donor chelate ligands.

## 2. Results and Discussion

**Scheme 1:**
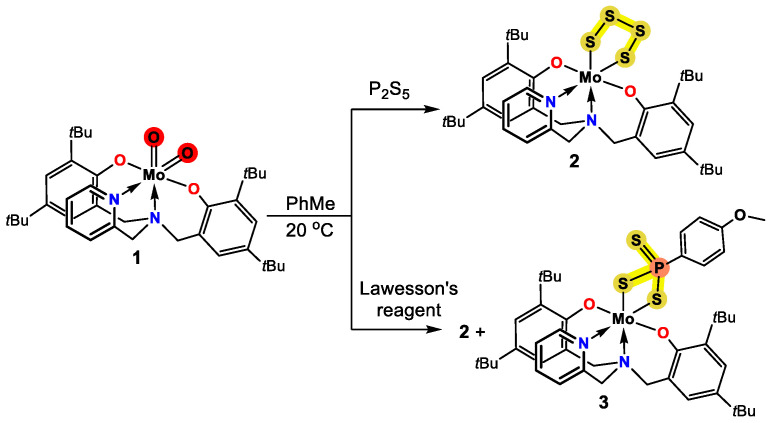
The reactions of a dioxidomolybdenum(VI) complex **1** with thionation reagents.

The oxido-to-sulfido substitution of dioxidomolybdenum(VI) aminobisphenolates was studied, allowing complex **1** to react with either P_2_S_5_ or Lawesson’s reagent in 1,2-dichloroethane at reflux temperature following the substitution procedure used previously to synthesize corresponding disulfidotungsten(VI) compounds. As a result, the reactions yielded dark mixtures of several strongly coloured, poorly stable compounds (seen by thin-layer chromatography), which could not be isolated or further characterized. However, **1** reacted with P_2_S_5_ at room temperature in a toluene solution to form a dark solution, which allowed the isolation of green **2** in ca. 15% yield (Scheme 1). The reaction was repeated using Lawesson‘s reagent under identical conditions, which again yielded **2** and brownish-red **3** along with several unstable compounds. The isolated yields of major products in the reaction with Lawesson‘s reagent depended on the reaction time—after a 20 h reaction, **3** was isolated in ca. 40% yield together with 5% of **2**, whereas **2** was found in ca. 35% yield with only a minor amount of **3** after one-week reaction.

**Figure 1:**
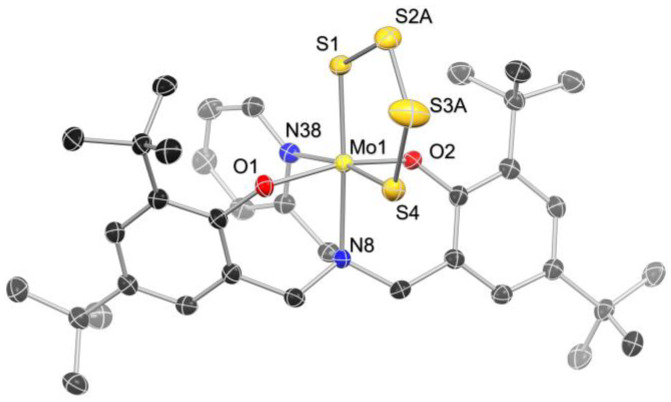
Molecular structure of **2**, with H-atoms and disorder of the S-atoms omitted. Displacement ellipsoids are drawn at the 50% probability level.

**Figure 2:**
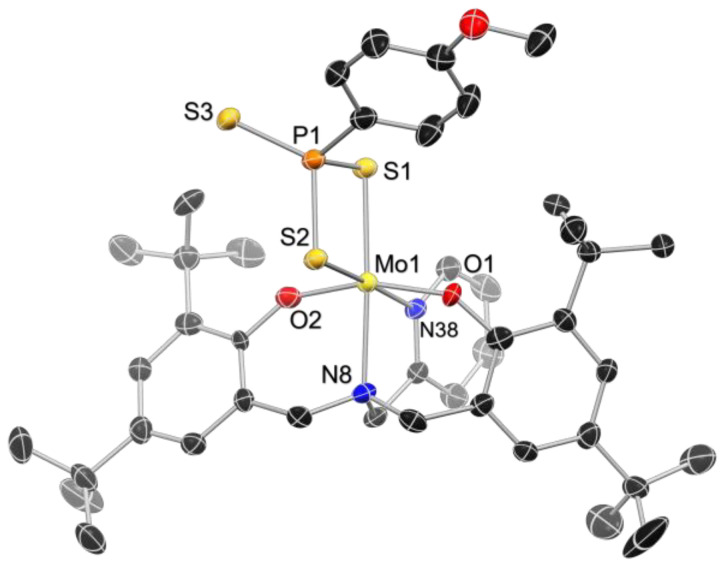
Molecular structure of **3**, with H-atoms and solvent molecules omitted. Displacement ellipsoids are drawn at the 50% probability level.

The molecular structures of **2** and **3** were studied by NMR spectroscopy and X-ray diffraction studies. The NMR spectra of **2** show the anticipated chemical shifts for the tetradentate aminobisphenolate ligand. For example, in the ^1^H NMR spectrum, the benzylic methylene protons are visible as two two-proton doublets at 5.66 and 4.30 ppm, respectively, accompanied by a two-proton singlet at 4.56 ppm for the methylene protons in the picoline arm. The XRD data shows that the product is virtually a *C*_s_ symmetric neutral molecule, where two oxides have been substituted by one S_4_^2−^ dianion to form a new Mo (IV) complex (Figure 1). The overall *cis*,*trans*,*cis* geometry of the parent complex **1** has been retained, i.e., the neutral nitrogen donors are in *cis* positions, phenolate oxygens are *trans* to each other and sulfur donors are *cis* (see Figure 1). Compared to **1**, the metal–donor distances to the aminobisphenolate ligand are somewhat shorter in **2,** whereas the O1-Mo-O2 and N8-Mo-N37 bite angles are slightly larger (see Table 1). The Mo-S distances and S-Mo-S angle are closely similar to the corresponding bonding parameters found previously for the dianionic tetrasulfido ligand in known Mo(IV) complexes [3,4,9,21,22]. In the same way, the ^1^H and ^13^C NMR spectra of **3** show chemical shifts for the coordinated aminobisphenolate ligand, while the number of the chemical shifts indicates a *C*_1_ symmetric structure. The spectra also show the presence of 4-methoxyphenyl group originated from Lawesson’s reagent. Correspondingly, the XRD studies show that the complex **3** is a neutral species, wherein one tetradentate aminobisphenolate and one bidentate, dianionic (4-methoxyphenyl) phosphonotrithioate surround the Mo(IV) ion (see Figure 2). The overall geometry around the metal centre is *cis,trans,cis* and the phenolate groups are in trans positions, i.e., the aminobisphenolate ligand is symmetrically coordinated. Therefore, the asymmetry seen in the NMR spectra rises from the phosphonotrithioate ligand. The metal–donor distances to the aminobisphenolate ligand are considerably shorter than in **2** (Table 1). The bite angles O1-Mo-O2 and N8-Mo-N37 are slightly larger and the S-Mo-S angle is clearly smaller than in **2**, obviously due to the rigidity of the phosphonotrithioate ligand. Complexes **2** and **3** are formally Mo(IV) complexes with d^2^ electron configurations, and are therefore expected to show paramagnetic properties caused by the two odd electrons. However, both compounds are diamagnetic, as evidenced by the well-resolved NMR spectra. Principally, the d^2^ metal centres may have either paramagnetic triplet or diamagnetic singlet electron configurations, depending on the coordination geometry. According to the visual inspection of the single crystal structures and further calculation of the continuous shape measures (CShM’s, see Table S2 Supplementary Materials) [23], **2** and **3** are octahedral complexes with axially compressed structures with four long bonds in xy-planes (Mo-N8, Mo-N37, Mo-S1 and Mo-S2/S4) and two shorter ones (Mo-O1 and Mo-O2) along z axes. This compression causes the splitting of the d_xy_, d_xz_ and d_yz_ orbital set, the first lower in energy compared to the other ones, which results singlet ground state system with both electrons on the d_xy_ orbital. The diamagnetic ground states of **2** and **3** were also confirmed by DFT calculations using the respective crystal structures of the complexes as the basis of coordinates for computational analyses. The singlet-triplet gap of **2** is 103 kJ/mol, whereas in **3** the triplet lies 35 kJ/mol above the singlet ground state, which affirms the singlet ground states of the complexes evident from the experimental data. For **3**, the lowest triplet state at the PBE0/def2-TZVP level shows Mo(IV) with d^2^ electron configuration, whereas for the lowest triplet state of **2**, the Mulliken spin densities (see ESI) in fact suggest a d^1^ Mo centre with the other unpaired electron localized at the S_4_-fragment while the d^2^ triplet state could not be established even with an attempt of using fragment-based initial assignment of partial charges and spins separately for ligands and metal.

Based on our investigation, it is evident that the reaction of Mo(VI) species **1** with thionation reagents leads to the formation new Mo(IV) compounds. Similar reactivity leading to the formation of Mo(IV) tetrasulfide complexes (Me_3_tacn)MoO(S_4_) and (Bu*^t^*_3_tach)MoO(S_4_) is seen in the reactions of Mo(VI) compounds (Me_3_tacn)MoO_3_ with S_8_ and (Bu*^t^*_3_tach)MoO_3_ with B_2_S_3_ (Me_3_tacn = 1,4,7-trimethyl-1,4,7-triazacyclonane, Bu*^t^*_3_tach = 1,3,5-tri-*tert*-butyl-1,3,5-triazacyclohexane) [3,9]. Although the formation mechanism of the reduced products **2** and **3** is not clear, we may suppose that the initial step is the oxido-to-sulfido substitution to form MoS_2_(L), similarly to the reactions of analogous W(VI) complexes. The unstable disulfido reduces then to eliminate elemental sulfur yielding a reactive Mo(IV) intermediate MoS(L). This intermediate reacts further with Lawesson’s reagent or other sulfur-containing species in the reaction mixture to form distinct isolatable products **2** and **3**. Both **2** and **3** are stable in the solid state as well as in inert solvents under open atmosphere. Although **3** decomposes in a prolonged reaction while **2** is formed, isolated and purified **3** is stable and can be stored in a toluene solution for a week without any noticeable formation of **2**. In general, the formation of these Mo(IV) complexes demonstrates the relative inclination of Mo(VI) toward reduction which is in stark contrast to the behaviour of W(VI), although these to M(VI) species often reflect each other’s reactivity.

In conclusion, the oxido-to-sulfido substitution of a dioxidomolybdenum(VI) aminobisphenolate **1** was studied using different thionation reagents. The reaction with P_2_S_5_ leads to the formation of Mo(IV) aminobisphenolate complex **2** with a bidentate sulfur chelate, i.e., tetrasulfide, S_4_^2−^. The parallel reaction with Lawesson’s reagent, the organic derivative of P_2_S_5_, yields also complex **2** together with complex **3** as the initial major product, of which the latter has a (4-methoxyphenyl)phosphonotrithioate residue derived from Lawesson’s reagent coordinated to the Mo(IV) centre. Both compounds **2** and **3** have an overall geometry of *cis*,*trans*,*cis*, that is, the neutral nitrogen donors are in *cis* positions, phenolate oxygen atoms are *trans* to each other and sulfur donors are *cis* in the axially compressed octahedral structures. Both **2** and **3** are stable in the solid state as well as in inert solvents under ambient atmosphere. The studied complexes are formally Mo(IV) with d^2^ metal centres. However, they are diamagnetic due to the singlet electron configurations as a result of the axially compressed octahedral structures. Similar thionation reactions of comparable dioxidotungsten(VI) aminobisphenolates are known to yield disulfidotungsten(VI) complexes, so these results demonstrate the higher inclination.

## 3. Materials and Methods

Complex **1** was prepared as reported previously [25]. Toluene was dried over 3A molecular sieves. Other chemicals were from commercial sources and were used as purchased. The synthetic reactions were run under a nitrogen atmosphere, whereas all isolations and analyses were conducted under open atmosphere. The IR spectra were measured using a Bruker VERTEX 70 FTIR instrument in transmittance mode, and peaks are reported in wavenumbers (cm^–1^) and intensities (b = broad, w = weak, m = medium, s = strong, vs = very strong). All NMR spectra were recorded on a Bruker Avance III 500 MHz instrument (^1^H: 500.08 MHz, ^13^C: 125.75 MHz) equipped with a broad-band smart probe and were referenced to residual CHCl_3_ solvent signals (^1^H: δ 7.26, ^13^C: δ 77.16). The NMR samples were kept under vacuum prior to the measurements to remove possible solvate molecules. The samples were dissolved in acetonitrile for mass spectrometric analysis. The mass spectra were obtained by quadrupole–Orbitrap mass spectrometer (QExactive^TM^, Thermo Fisher Scientific GmbH, Bremen, Germany) using direct infusion, negative electrospray ionization and full scan at *m*/*z* 150–2000 with the resolution of 140,000. The calibration was performed by Pierce ESI Negative Ion Calibration Solution (Thermo Fisher Scientific Inc., Waltham, MA, USA). The data was processed with Thermo Xcalibur Qual Browser software (Version 3.0.63, Thermo Fisher Scientific Inc., Waltham, MA, USA). Single crystal X-ray diffraction data were collected with Rigaku Oxford Diffraction custom system consisting of microfocus MicroMax™-007 HF rotating anode generator producing monochromatized Cu K_α1_ radiation and HyPix-6000HE detector. Data collection and reduction were done using the CrysAlis^Pro^ software [26], whereas crystal structures were solved and refined using SHELXS and SHELXL programs within the Olex^2^ interface. DFT calculations were conducted using Gaussian 16 software [27]. The geometries were taken from single crystal X-ray structures and the C–H bond lengths were normalized. The relative energies of singlet and triplet spin states were determined as single point calculations at the PBE0/def2-TZVP (effective core potential for Mo) level using these geometries [28,29].

**2**: *Method a:* First, 0.35 g (0.50 mmol) of **1** and 0.15 g (0.68 mmol) of P_2_S_5_ were mixed in 20 mL of dry toluene. The dark mixture was stirred for 24 h, the solvent was evaporated and the green product (60 mg, 15%) was isolated by a silica column chromatography using CH_2_Cl_2_ as an eluent. *Method b*: First, 0.50 g (0.75 mmol) of **1** and 0.61 g (1.50 mmol) of Lawesson’s reagent were mixed in 20 mL of dry toluene and the solution was kept for week at room temperature. Afterwards, 132 mg (35%) of **2** as well as 33 mg (5%) of **3** were isolated by column chromatography using CH_2_Cl_2_ as an eluent. The crystals of **2** for XRD analyses were grown from acetonitrile at room temperature. IR: 3440 s, 2958 s, 2868 s, 1626 s (br), 1606 s, 1470 s, 1443 s, 1412 m, 1390 m, 1362 m, 1304 m, 1259 s, 1240 s, 1203 m, 1171 m, 1128 m, 1057 w, 1022 w, 976 w, 914 m, 850 s, 808 m, 760 s, 727 w, 648 w, 590 w, 557 s, 509 w, 474 m cm^−1^. UV-Vis: λ = 425 nm, ε = 7062 cm^−1^M^−1^, ^1^H NMR (CDCl_3_): 10.11 (1H, d, *J* = 5.3 Hz, ArH), 7.69 (1H, t, *J* = 8.0 Hz, ArH), 7.38 (1H, t, *J* = 6.2 Hz, ArH), 7.09 (1H, d, *J* = 7.8 Hz, ArH), 7.02 (2H, d, *J* = 2.0 Hz, ArH), 6.87 (2H, d, *J* = 2.1 Hz, ArH), 5.66 (2H, d, *J* = 13.4 Hz, CH_2_), 4.56 (2H, CH_2_), 4.30 (2H, d, *J* = 13.4 Hz, CH_2_), 1.22 (18H, *t*Bu), 0.56 (18H, *t*Bu). ^13^C NMR (CDCl_3_): 158.28, 157.92, 154.54, 141.15, 138.43, 135.02, 123.09, 122.51, 122.44, 121.19, 120.57, 66.41, 61.59, 33.23, 33.15, 30.54, 28.68, 28.13. ESI(-)-MS: (MeCN): *m*/*z* = 767.17367 [M-H]^−^ (calcd. *m*/*z* = 767.17310).

**3**: First, 3.9 g (5.8 mmol) of **1** and 2.4 g (5.9 mmol) of Lawesson’s reagent were mixed in 35 mL of dry toluene and the reaction mixture was stirred under a nitrogen atmosphere for 20 h at room temperature. Next, 3.55 g of brown precipitate was isolated and recrystallized from hot acetonitrile to obtain 2.08 g (39%) of brownish-red crystals. The filtrate was evaporated and 0.22 g (5%) of complex **2** was isolated by column chromatography. IR: 3440 s, 2958 s, 1622 s (br), 1605 s, 1593 s, 1570 m, 1497 s, 1475 s, 1443 s, 1414 m, 1392 m, 1362 m, 1302 w, 1288 w, 1250 s, 1203 m, 1171 m, 1126 m, 1095 m, 1055 w, 1022 m, 916 m, 868 s, 854 s, 808 w, 798 w, 762 s, 667 m, 619 m, 594 w, 575 m, 559 w, 532 w, 507 w cm^−1^. UV-Vis: λ = 408 nm, ε= 4408 cm^−1^M^−1^. ^1^H NMR (CDCl_3_): 8.90 (1H, d, *J* = 5.1 Hz, ArH), 8.43 (2H, dd, *J* = 12.1 Hz, *J*’ = 11.7 Hz, ArH), 7.62 (1H, t, *J* = 7.8 Hz, ArH), 7.29 (1H, t, 7.8 Hz, ArH), 7.09 (1H, d, *J* = 6.2 Hz, ArH), 7.08 (1H, d, *J* = 6.3 Hz, ArH), 7.04 (2H, t, *J* = 2.5 Hz, ArH), 6.95 (1H, d, *J* = 7.9 Hz, ArH), 6.91 (2H, d, *J* = 6.7 Hz, ArH), 4.83 (1H, d, *J* = 13.1 Hz, CH), 4.66 (1H, d, *J* = 12.7 Hz, CH), 4.54 (1H, d, *J* = 17.0 Hz, CH), 4.36 (1H, d, *J* = 17.2 Hz, CH), 4.22 (1H, d, *J* = 13.2 Hz, CH), 4.12 (1H, d, *J* = 12.7 Hz, CH), 3.81 (3H, OCH_3_), 1.24 (18H, _t_BuH), 1.05 (9H, *t*BuH), 0.86 (9H, *t*BuH) ppm. ^13^C NMR (CDCl_3_): 161.30, 161.27, 160.61, 157.19, 156.97, 154.47, 144.46, 144.30, 139.91, 136.75, 135.95, 132.33 132.20, 125.10, 124.75, 124.65, 124.53, 123.84, 123.40, 123.17, 120.55, 113.46, 113.34, 67.07, 66.65, 60.90, 55.32, 34.92, 34.68, 34.60, 34.53, 31.45, 31.44, 29.90, 29.83 ppm. ESI(-)-MS: (MeCN): *m*/*z* = 873.22504 [M-H]^−^ (calcd. *m*/*z* = 873.22448).

## Figures and Tables

**Table 1:** Selected bond lengths (Å) and angles (°) for studied complexes.

	1 [24]	2	3
Mo-O1	1.951 (3)	1.9370 (15)	1.901 (6)
Mo-O2	1.951 (4)	1.9152 (15)	1.883 (6)
Mo-N8	2.372 (3)	2.3388 (19)	2.290 (7)
Mo-N38	2.345 (4)	2.309 (2)	2.237 (6)
Mo-S1		2.2254 (6)	2.378 (2)
Mo-S2/S4		2.2223 (6)	2.373 (2)
O1-Mo-O2	156.5 (1)	158.01 (7)	161.5 (3)
N8-Mo-N38	68.9 (1)	73.89 (7)	75.9 (3)
S1-Mo-S2/S4		95.62 (2)	80.71 (7)
N8-Mo-S1		167.29 (5)	175.0 (3)
N37-Mo-S2/S4		94.05 (5)	101.76 (19)

## Data Availability

CCDC 2177727 and 2177728 contain the supplementary crystallographic data for this paper. These data can be obtained free of charge from The Cambridge Crystallographic Data Centre via www.ccdc.cam.ac.uk/structures (accessed on 21 October 2022).

## Supplementary Materials

The following supporting information can be downloaded at: https://www.mdpi.com/article/10.3390/molecules27217154/s1, Electronic Supplementary Information (ESI) available: Details of computational methods and crystallographic data.

## Sample Availability

Samples of the compounds **1–3** are available from the authors.
