# Supplementary material for: Reactions of a Dioxidomolybdenum(VI) Complex with Thionation Reagents—Formation of Mo(IV) Species with Sulfur Donors
Source: Molecules. 2022 Oct 22;27(21):7154. doi: 10.3390/molecules27217154 (PMC9655758; doi:10.3390/molecules27217154)

checkCIF/PLATON report

Structure factors have been supplied for datablock(s) 3

THIS REPORT IS FOR GUIDANCE ONLY. IF USED AS PART OF A REVIEW PROCEDURE FOR PUBLICATION, IT SHOULD NOT REPLACE THE EXPERTISE OF AN EXPERIENCED CRYSTALLOGRAPHIC REFEREE.

No syntax errors found. CIF dictionary Interpreting this report

Datablock: 3

Bond precision:	C-C = 0.0139 A		Wavelength=1.54184
Cell:	a=26.8402(17)	b=18.5045(15)	c=9.4729(7)
	alpha=90	beta=90	gamma=90
Temperature:	125 K		

	Calculated	Reported
Volume	4704.9(6)	4704.8(6)
Space group	P 21 21 2	P 21 21 2
Hall group	P 2 2ab	P 2 2ab
Moiety formula	C43 H57 Mo N2 O3 P S3, C2 H3 N	C43 H57 Mo N2 O3 P S3, C2 H3 N1
Sum formula	C45 H60 Mo N3 O3 P S3	C45 H60 Mo N3 O3 P S3
Mr	914.05	914.05
Dx,g cm-3	1.290	1.290
Z	4	4
Mu (mm-1)	4.158	4.158
F000	1920.0	1920.0
F000’	1928.92	
h,k,lmax	32,22,11	32,22,11
Nref	8621[4826]	7781
Tmin,Tmax	0.670,0.847	0.210,1.000
Tmin’	0.274	

Correction method= # Reported T Limits: Tmin=0.210 Tmax=1.000
AbsCorr = GAUSSIAN

Data completeness= 1.61/0.90 Theta(max)= 68.251

R(reflections)= 0.0585(6707) wR2(reflections)= 0.1704(7781)

S = 1.060 Npar= 548

The following ALERTS were generated. Each ALERT has the format
test-name_ALERT_alert-type_alert-level.
Click on the hyperlinks for more details of the test.

Alert level C

```
PLAT042_ALERT_1_C Calc. and Reported MoietyFormula Strings  Differ     Please Check
PLAT213_ALERT_2_C Atom C23              has ADP max/min Ratio .....        3.4 prolat
PLAT220_ALERT_2_C NonSolvent   Resd 1  C   Ueq(max)/Ueq(min) Range        5.1 Ratio
PLAT222_ALERT_3_C NonSolvent Resd 1  H   Uiso(max)/Uiso(min) Range        5.7 Ratio
PLAT242_ALERT_2_C Low    'MainMol' Ueq as Compared to Neighbors of        C20 Check
PLAT342_ALERT_3_C Low Bond Precision on  C-C Bonds ...............    0.01393 Ang.
PLAT911_ALERT_3_C Missing FCF Refl Between Thmin & STh/L=    0.600         47 Report
PLAT915_ALERT_3_C No Flack x Check Done: Low Friedel Pair Coverage         79 %
PLAT971_ALERT_2_C Check Calcd Resid. Dens.  1.14Ang From N38             1.55 eA-3
PLAT971_ALERT_2_C Check Calcd Resid. Dens.  0.99Ang From Mo1             1.52 eA-3
PLAT975_ALERT_2_C Check Calcd Resid. Dens.  0.61Ang From O1      .       0.61 eA-3
PLAT975_ALERT_2_C Check Calcd Resid. Dens.  0.73Ang From O2      .       0.56 eA-3
PLAT977_ALERT_2_C Check Negative Difference Density on H21B      .      -0.45 eA-3
```

Alert level G

```
PLAT002_ALERT_2_G Number of Distance or Angle Restraints on AtSite          9 Note
PLAT003_ALERT_2_G Number of Uiso or Uij Restrained non-H Atoms ...          7 Report
PLAT072_ALERT_2_G SHELXL First  Parameter in WGHT  Unusually Large       0.10 Report
PLAT171_ALERT_4_G The CIF-Embedded .res File Contains EADP Records          2 Report
PLAT172_ALERT_4_G The CIF-Embedded .res File Contains DFIX Records          6 Report
PLAT186_ALERT_4_G The CIF-Embedded .res File Contains ISOR Records          3 Report
PLAT300_ALERT_4_G Atom Site Occupancy of N46A       Constrained at        0.7 Check
PLAT300_ALERT_4_G Atom Site Occupancy of C47A       Constrained at        0.7 Check
PLAT300_ALERT_4_G Atom Site Occupancy of C48A       Constrained at        0.7 Check
PLAT300_ALERT_4_G Atom Site Occupancy of H48A       Constrained at        0.7 Check
PLAT300_ALERT_4_G Atom Site Occupancy of H48B       Constrained at        0.7 Check
PLAT300_ALERT_4_G Atom Site Occupancy of H48C       Constrained at        0.7 Check
PLAT300_ALERT_4_G Atom Site Occupancy of N46B       Constrained at       0.15 Check
PLAT300_ALERT_4_G Atom Site Occupancy of C47B       Constrained at       0.15 Check
PLAT300_ALERT_4_G Atom Site Occupancy of C48B       Constrained at       0.15 Check
PLAT300_ALERT_4_G Atom Site Occupancy of H48G       Constrained at       0.15 Check
PLAT300_ALERT_4_G Atom Site Occupancy of H48H       Constrained at       0.15 Check
PLAT300_ALERT_4_G Atom Site Occupancy of H48I       Constrained at       0.15 Check
PLAT300_ALERT_4_G Atom Site Occupancy of N46C       Constrained at       0.15 Check
PLAT300_ALERT_4_G Atom Site Occupancy of C47C       Constrained at       0.15 Check
PLAT300_ALERT_4_G Atom Site Occupancy of C48C       Constrained at       0.15 Check
PLAT300_ALERT_4_G Atom Site Occupancy of H48D       Constrained at       0.15 Check
PLAT300_ALERT_4_G Atom Site Occupancy of H48E       Constrained at       0.15 Check
PLAT300_ALERT_4_G Atom Site Occupancy of H48F       Constrained at       0.15 Check
PLAT302_ALERT_4_G Anion/Solvent/Minor-Residue Disorder (Resd  2  )       100% Note
PLAT302_ALERT_4_G Anion/Solvent/Minor-Residue Disorder (Resd  3  )       100% Note
PLAT302_ALERT_4_G Anion/Solvent/Minor-Residue Disorder (Resd  4  )       100% Note
PLAT304_ALERT_4_G Non-Integer Number of Atoms in ..... (Resd  2  )       4.20 Check
PLAT304_ALERT_4_G Non-Integer Number of Atoms in ..... (Resd  3  )       0.90 Check
PLAT304_ALERT_4_G Non-Integer Number of Atoms in ..... (Resd  4  )       0.90 Check
PLAT413_ALERT_2_G Short Inter XH3 .. XHn     H23C     ..H48F     .       1.82 Ang.
                                          1/2-x,-1/2+y,2-z  =      4_547 Check
PLAT432_ALERT_2_G Short Inter X...Y Contact  C23      ..C48C     .       3.14 Ang.
                                          1/2-x,-1/2+y,2-z  =      4_547 Check
```

```
PLAT432_ALERT_2_G Short Inter X...Y Contact  C35      ..C48C     .       3.08 Ang.
                                                     x,y,z  =      1_555 Check
PLAT432_ALERT_2_G Short Inter X...Y Contact  C36      ..C48C     .       3.18 Ang.
                                                     x,y,z  =      1_555 Check
PLAT789_ALERT_4_G Atoms with Negative _atom_site_disorder_group  #         12 Check
PLAT794_ALERT_5_G Tentative Bond Valency for Mo1       (V)       .       4.67 Info
PLAT860_ALERT_3_G Number of Least-Squares Restraints ............          48 Note
PLAT933_ALERT_2_G Number of HKL-OMIT Records in Embedded .res File          4 Note
PLAT941_ALERT_3_G Average HKL Measurement Multiplicity ..........         3.5 Low
PLAT978_ALERT_2_G Number C-C Bonds with Positive Residual Density.          0 Info
```

```
   0 ALERT level A = Most likely a serious problem - resolve or explain
   0 ALERT level B = A potentially serious problem, consider carefully
  13 ALERT level C = Check. Ensure it is not caused by an omission or oversight
  40 ALERT level G = General information/check it is not something unexpected

   1 ALERT type 1 CIF construction/syntax error, inconsistent or missing data
  17 ALERT type 2 Indicator that the structure model may be wrong or deficient
   6 ALERT type 3 Indicator that the structure quality may be low
  28 ALERT type 4 Improvement, methodology, query or suggestion
   1 ALERT type 5 Informative message, check
```

It is advisable to attempt to resolve as many as possible of the alerts in all categories. Often the minor alerts point to easily fixed oversights, errors and omissions in your CIF or refinement strategy, so attention to these fine details can be worthwhile. In order to resolve some of the more serious problems it may be necessary to carry out additional measurements or structure refinements. However, the purpose of your study may justify the reported deviations and the more serious of these should normally be commented upon in the discussion or experimental section of a paper or in the "special_details" fields of the CIF. checkCIF was carefully designed to identify outliers and unusual parameters, but every test has its limitations and alerts that are not important in a particular case may appear. Conversely, the absence of alerts does not guarantee there are no aspects of the results needing attention. It is up to the individual to critically assess their own results and, if necessary, seek expert advice.

Publication of your CIF in IUCr journals

A basic structural check has been run on your CIF. These basic checks will be run on all CIFs submitted for publication in IUCr journals (*Acta Crystallographica*, *Journal of Applied Crystallography*, *Journal of Synchrotron Radiation*); however, if you intend to submit to *Acta Crystallographica Section C* or *E* or *IUCrData*, you should make sure that full publication checks are run on the final version of your CIF prior to submission.

Publication of your CIF in other journals

Please refer to the *Notes for Authors* of the relevant journal for any special instructions relating to CIF submission.

PLATON version of 18/05/2022; check.def file version of 17/05/2022

Datablock 3 - ellipsoid plot

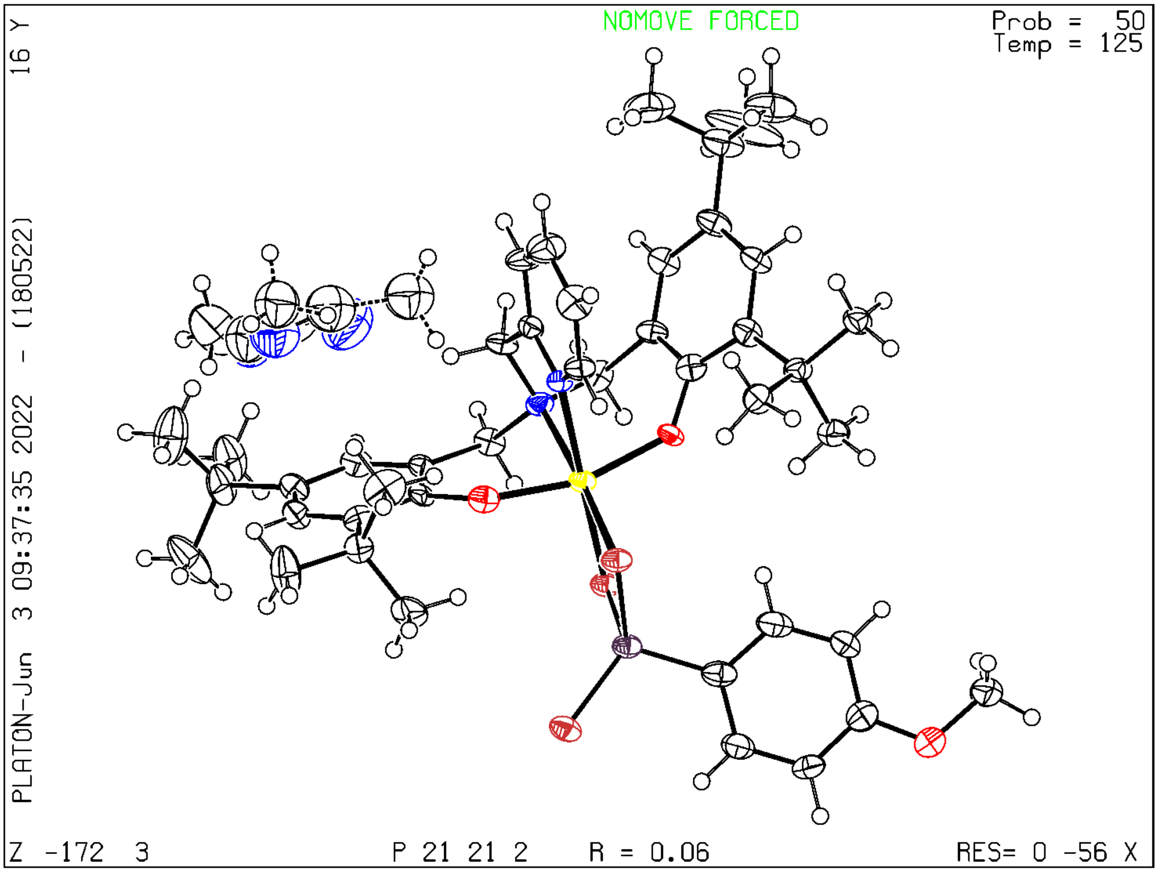

Supplement: Supplementary file 1 [file molecules-27-07154-s001.zip › 3_checkcif.pdf]
