# Supplementary material for: Reactions of a Dioxidomolybdenum(VI) Complex with Thionation Reagents—Formation of Mo(IV) Species with Sulfur Donors
Source: Molecules. 2022 Oct 22;27(21):7154. doi: 10.3390/molecules27217154 (PMC9655758; doi:10.3390/molecules27217154)
Supplement: Supplementary file 1 [file molecules-27-07154-s001.zip › molecules-1978680-Supporting information.pdf]

Supporting information for:

Reactions of a dioxidomolybdenum(VI) complex with thionation reagents - formation of Mo(IV) species with sulfur donors

Esko Salojärvi, [a] Anssi Peuronen, [a] Narhari Sapkota[a] and Ari Lehtonen [*a]

[a]Intelligent Materials Chemistry group, Department of Chemistry, University of Turku, FI-20014 Turku, Finland

Content:

Table S1. Crystallographic data for complexes **2** and **3**.

Table S2. Shape measures of complexes **2** and **3** for ML_6 structures.

Table S3. Calculated single point energies for complexes **2** and **3** (PBE0/def2-TZVP).

Figure S1. Selected Mulliken spin populations for the excited triplet states of complexes.

Table S4. xyz-coordinates for DFT calculations.

Figure S2. The ^{1}H spectrum of **2**.

Figure S3. The ^{13}C spectrum of **2**.

Figure S4. The ^{1}H spectrum of **3**.

Figure S5. The ^{13}C NMR spectrum of **3.**

Table S1. Crystallographic data for complexes **2** and **3**.

Identification code	**2**	**3**
Empirical formula	$C_{36}H_{50}MoN_2O_2S_4$	$C_{45}H_{60}MoN_3O_3PS_3$
Formula weight	766.96	914.05
Temperature/K	125.0(1)	125.0(1)
Crystal system	triclinic	orthorhombic
Space group	*P*–1	$P2_12_12$
a/Å	10.0273(2)	26.8402(17)
b/Å	10.0625(3)	18.5045(15)
c/Å	19.4957(3)	9.4729(7)
α/°	101.1045(17)	90
β/°	96.0498(14)	90
γ/°	101.0893(19)	90
Volume/Å^3	1873.41(7)	4704.8(6)
Z	2	4
ρ_{calc}g/cm^3	1.36	1.29
μ/mm^{-1}	5.199	4.158
F(000)	804	1920
Crystal size/mm^3	0.22 × 0.19 × 0.16	0.30 × 0.09 × 0.04
Radiation	Cu Kα (λ = 1.54184)	Cu Kα (λ = 1.54184)
2Θ range for data collection/°	4.67 to 144.234	5.802 to 136.502
Index ranges	-12 ≤ h ≤ 12, -12 ≤ k ≤ 12, -17 ≤ l ≤ 24	-32 ≤ h ≤ 32, -21 ≤ k ≤ 22, -11 ≤ l ≤ 11
Reflections collected	21711	16605
Independent reflections	7327 [R_{int} = 0.0562, R_{sigma} = 0.0476]	7781 [R_{int} = 0.0836, R_{sigma} = 0.1003]
Data/restraints/parameters	7327/0/437	7781/48/548
Goodness-of-fit on F^2	1.036	1.06
Final R indexes [I>=2σ (I)]	R_1 = 0.0363, wR_2 = 0.0966	R_1 = 0.0585, wR_2 = 0.1528
Final R indexes [all data]	R_1 = 0.0381, wR_2 = 0.0979	R_1 = 0.0705, wR_2 = 0.1704
Largest diff. peak/hole / e Å^{-3}	0.89/-0.77	1.50/-1.02
Flack parameter		-0.026(13)

Table S2. Shape measures of complexes **2** and **3** for ML_6 structures.[a]

	HP-6	PPY-6	OC-6	TPR-6	JPPY-6
**2**	31.233	27.116	1.476	15.507	29.626
**3**	28.892	25.782	2.306	15.350	28.055

[a] HP-6 = D_{6h} hexagon, PPY-6 = C_{5v} pentagonal pyramid, OC-6 = O_h octahedron, TPR-6 = D_{3h} trigonal prism, JPPY-6 = C_{5v} Johnson pentagonal pyramid J2

Table S3. Calculated single point energies for complexes **2** and **3** (PBE0/def2-TZVP).

	Singlet (E_h)	Triplet (E_h)	Singlet-triplet gap (kJ mol^{-1})
**2**	-3321.194852	-3321.155574	103.1
**3**	-3610.295090	-3610.281677	35.2

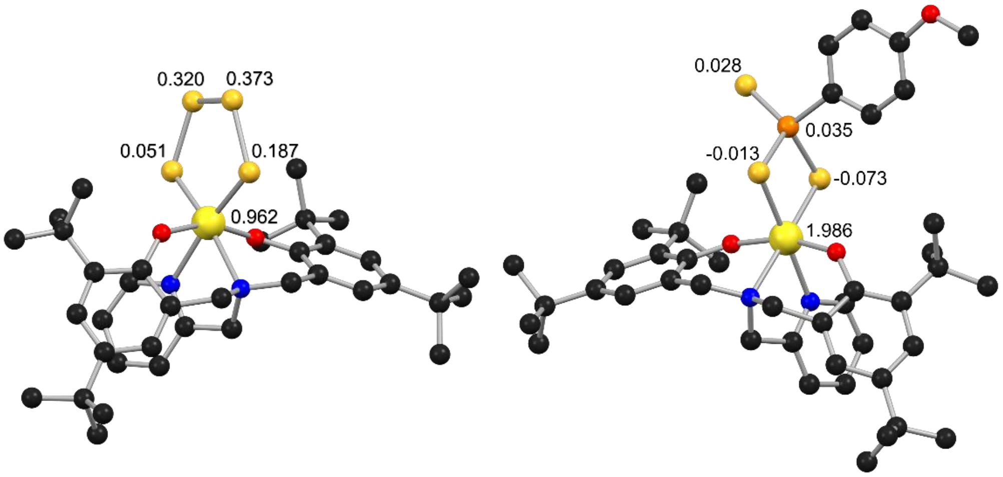

Figure S1. Selected Mulliken spin populations for the excited triplet states of complexes complex **2** (left) and **3** (right).

Table S4. xyz-coordinates for DFT calculations.

Complex **2**

Mo	2.799682	0.788699	4.673113
S	4.765659	1.307059	3.768157
S	3.486921	-1.101578	5.617990
S	6.315852	-0.150734	4.018662
S	5.649959	-1.467515	5.363891
O	2.892569	1.887034	6.265820
O	2.032169	-0.020164	3.115976
N	0.551577	0.593532	5.287832
N	1.755441	2.696396	3.898140
C	2.074697	2.754464	6.898702
C	2.547392	3.978135	7.401413
C	1.585080	4.863838	7.892016
H	1.917312	5.835275	8.255131
C	0.215442	4.572968	7.943100
C	-0.184081	3.301535	7.548045
H	-1.232118	3.018409	7.633936
C	0.729290	2.387715	7.046280
C	0.339213	0.973463	6.720284
H	0.915900	0.297566	7.349980
H	-0.712677	0.835334	6.965985
C	0.093432	-0.817701	5.142714
H	-0.937810	-0.880189	5.487032
H	0.695375	-1.443760	5.799703
C	0.163352	-1.387107	3.745075
C	-0.774836	-2.344807	3.368940
H	-1.538891	-2.646002	4.084078
C	-0.764491	-2.927268	2.109230
C	0.230458	-2.525722	1.222816
H	0.263665	-2.994903	0.240631
C	1.190689	-1.550161	1.523459
C	1.139985	-0.987614	2.810414
C	4.051329	4.302453	7.452498
C	4.311652	5.579368	8.260393
H	3.877700	5.475310	9.253760
H	3.856714	6.428790	7.753012
H	5.384911	5.741647	8.348154
C	4.808250	3.153606	8.134762
H	4.688833	2.242835	7.549821
H	4.405935	2.997723	9.134645
H	5.865570	3.405115	8.203602
C	4.605829	4.516030	6.033101
H	5.653159	4.808488	6.092185
H	4.037348	5.301141	5.536764
H	4.517669	3.590625	5.465851
C	-0.824288	5.607560	8.394916
C	-1.635670	5.058786	9.568917
H	-2.389004	5.788365	9.862384
H	-0.971979	4.864019	10.410048
H	-2.124640	4.132587	9.270612
C	-0.197599	6.928836	8.814378
H	-0.982808	7.634945	9.080420
H	0.387646	7.331532	7.989002
H	0.451181	6.766798	9.673883
C	-1.761899	5.886176	7.201426
H	-2.296637	4.974376	6.939517
H	-1.174342	6.220466	6.347642
H	-2.477485	6.660168	7.474911
C	-1.836413	-3.962218	1.716445
C	-1.741676	-4.371089	0.245585
H	-1.848836	-3.488891	-0.383831
H	-2.534184	-5.081104	0.013789
H	-0.773563	-4.833730	0.059463
C	-1.672306	-5.216680	2.588480
H	-0.678476	-5.635818	2.438326
H	-2.422743	-5.954531	2.308585
H	-1.799133	-4.950000	3.636677
C	-3.241730	-3.394940	1.957300
H	-3.352027	-3.133798	3.008756
H	-3.986354	-4.142707	1.688412
H	-3.383149	-2.505012	1.345785
C	2.264307	-1.131284	0.506495
C	2.277272	0.385805	0.292886
H	1.277276	0.723085	0.024241
H	2.971599	0.633312	-0.508720
H	2.592274	0.878755	1.211414
C	3.647262	-1.592576	1.006368
H	3.848793	-1.147210	1.979485
H	4.412453	-1.279418	0.297612
H	3.657495	-2.677909	1.095073
C	2.018747	-1.773504	-0.865791
H	2.026570	-2.857909	-0.766157
H	2.804040	-1.466564	-1.555011
H	1.052336	-1.450470	-1.250004
C	-0.266395	1.477710	4.420149
H	-1.215338	1.683121	4.913336
H	-0.480211	0.961710	3.485298
C	0.420291	2.771294	4.119696
C	-0.278044	3.961960	4.019040
H	-1.348381	3.986026	4.218342
C	0.390100	5.121664	3.663718
H	-0.142724	6.067645	3.579222
C	1.754138	5.050204	3.417944
H	2.314048	5.939215	3.131445
C	2.389636	3.828324	3.543385
H	3.459308	3.780513	3.344799

Complex **3**

Mo	6.033677	8.230802	5.352662
S	5.004087	8.386795	3.215102
S	5.690659	10.577172	5.263143
S	5.907260	11.462798	1.924893
P	4.928398	10.481689	3.308884
O	4.482313	7.712676	6.321266
O	7.775606	8.227101	4.636037
O	-0.799838	12.172260	3.244468
N	6.999924	7.990243	7.356654
N	6.559745	6.004710	5.456390

C	4.197807	6.787451	7.271398
C	4.605778	4.522500	8.036808
H	5.003365	3.524513	7.858295
C	3.465070	7.090924	8.436565
C	3.406021	6.060224	9.397117
H	2.857384	6.257883	10.316817
C	3.996506	4.809320	9.253129
C	4.710455	5.471781	7.067731
C	5.343884	5.181260	5.715001
H	5.612085	4.126835	5.668338
H	4.611139	5.372714	4.932472
C	7.107285	5.532845	4.136815
H	6.391705	5.776241	3.352835
H	7.209754	4.449081	4.166394
C	8.446611	6.141644	3.782529
C	9.428962	5.373707	3.139319
H	9.227433	4.320030	2.952044
C	10.623351	5.891833	2.738615
C	10.872965	7.238960	3.044590
H	11.832728	7.663507	2.753838
C	9.955030	8.066112	3.701062
C	8.723065	7.472117	4.028824
C	7.563568	5.762301	6.516408
H	8.560635	5.820940	6.082427
H	7.430837	4.756007	6.910964
C	7.458892	6.754143	7.646525
C	7.016028	8.906216	8.328574
H	6.672720	9.911858	8.090365
C	7.437419	8.656405	9.606468
H	7.412554	9.435428	10.367011
C	7.885651	7.403650	9.900128
H	8.230666	7.171999	10.906718
C	7.904439	6.422912	8.920630
H	8.258058	5.417439	9.144009
C	2.726964	8.404744	8.632654
C	1.924442	8.443603	9.948440
H	2.608890	8.383203	10.793309
H	1.234880	7.601276	9.978599
H	1.363010	9.375082	10.003785
C	3.703948	9.594583	8.661072
H	3.157816	10.505736	8.900782
H	4.175016	9.701462	7.685064
H	4.468222	9.420207	9.416981
C	1.723141	8.584238	7.462751
H	1.016604	7.755550	7.460969
H	2.266500	8.602079	6.519160
H	1.184947	9.522529	7.588755
C	3.975034	3.769367	10.373773
C	5.443193	3.356716	10.661749
H	5.462987	2.634994	11.477007

H	6.019405	4.237318	10.941821
H	5.874946	2.907267	9.768718
C	3.497278	4.341156	11.695242
H	2.457130	4.649840	11.601822
H	4.108311	5.203012	11.959372
H	3.582912	3.582768	12.472054
C	3.247664	2.542518	9.951281
H	3.707691	2.141438	9.049378
H	2.206138	2.788162	9.749281
H	3.298747	1.798667	10.745005
C	11.729167	5.062831	2.022464
C	12.102246	5.751199	0.709520
H	11.212236	5.862843	0.091994
H	12.838882	5.149166	0.179571
H	12.520645	6.734064	0.921270
C	12.963817	4.990664	2.909128
H	13.353705	5.995011	3.067854
H	13.722392	4.377472	2.424896
H	12.695242	4.549873	3.868029
C	11.259464	3.660190	1.691860
H	10.977566	3.145668	2.609314
H	12.065911	3.114407	1.204331
H	10.400741	3.711100	1.024084
C	10.269061	9.539070	4.029772
C	10.212696	9.733367	5.555856
H	9.205729	9.520854	5.911914
H	10.473059	10.761964	5.801051
H	10.917438	9.055500	6.035185
C	11.635227	9.955421	3.537181
H	12.400955	9.415411	4.092132
H	11.764852	11.026335	3.686383
H	11.724477	9.723246	2.476969
C	9.233029	10.464295	3.378983
H	9.253846	10.331058	2.298365
H	9.466763	11.499949	3.621285
H	8.240408	10.220181	3.754528
C	3.199352	11.008327	3.331619
C	2.335097	10.630835	4.363218
H	2.730131	10.037734	5.186687
C	1.001139	10.980570	4.374585
H	0.356270	10.664608	5.193263
C	0.493860	11.742956	3.329724
C	1.323222	12.144503	2.294336
H	0.923413	12.743975	1.477815
C	2.659864	11.785516	2.296231
H	3.302886	12.111728	1.480125
C	-1.707037	11.705947	4.235334
H	-2.711322	12.056312	4.001720
H	-1.407790	12.085147	5.211335
H	-1.698691	10.617083	4.250430

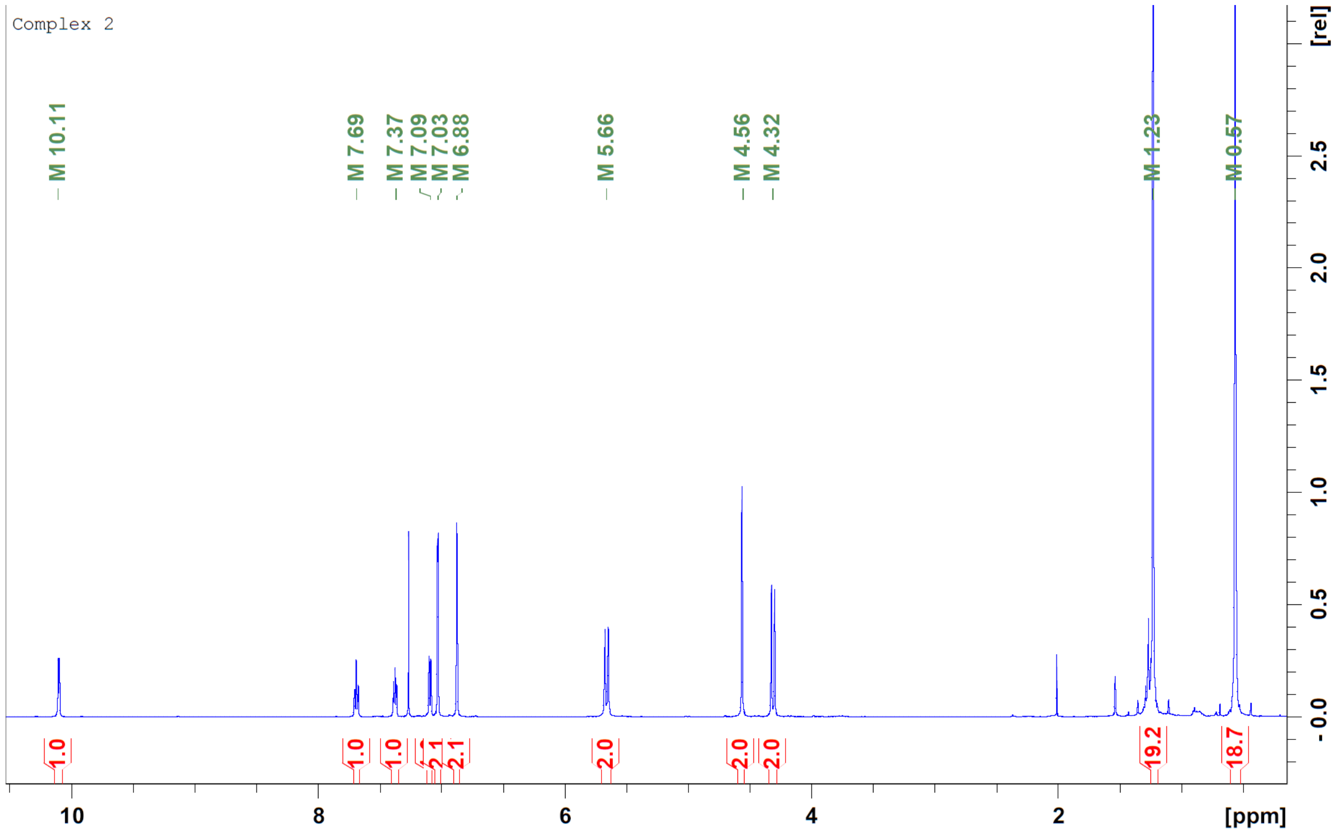

Figure S2. The ^{1}H spectrum of **2**. Peaks at 2.1 and 1.56 ppm are due to the acetonitrile of crystallization and water impurity in the NMR solvent, respectively.

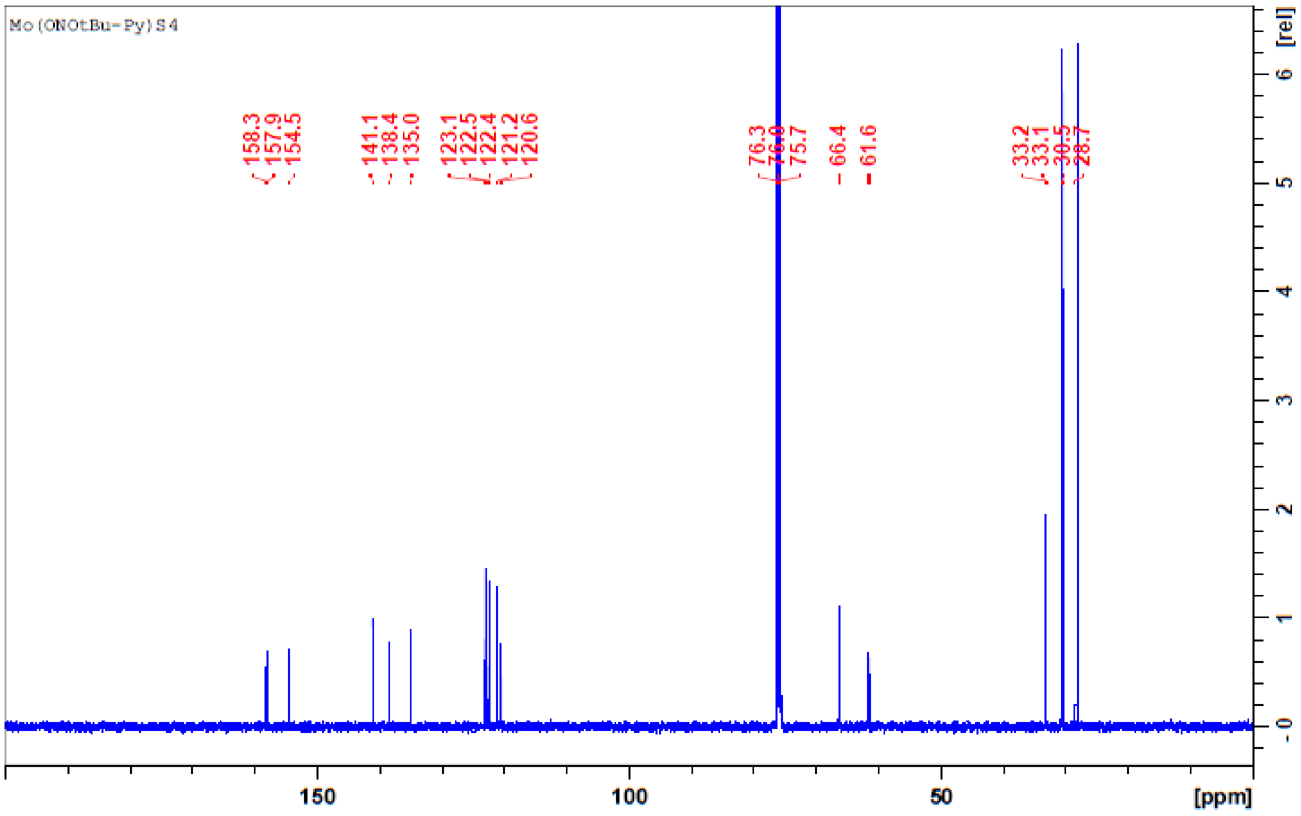

Figure S3. The ^{13}C spectrum of **2**.

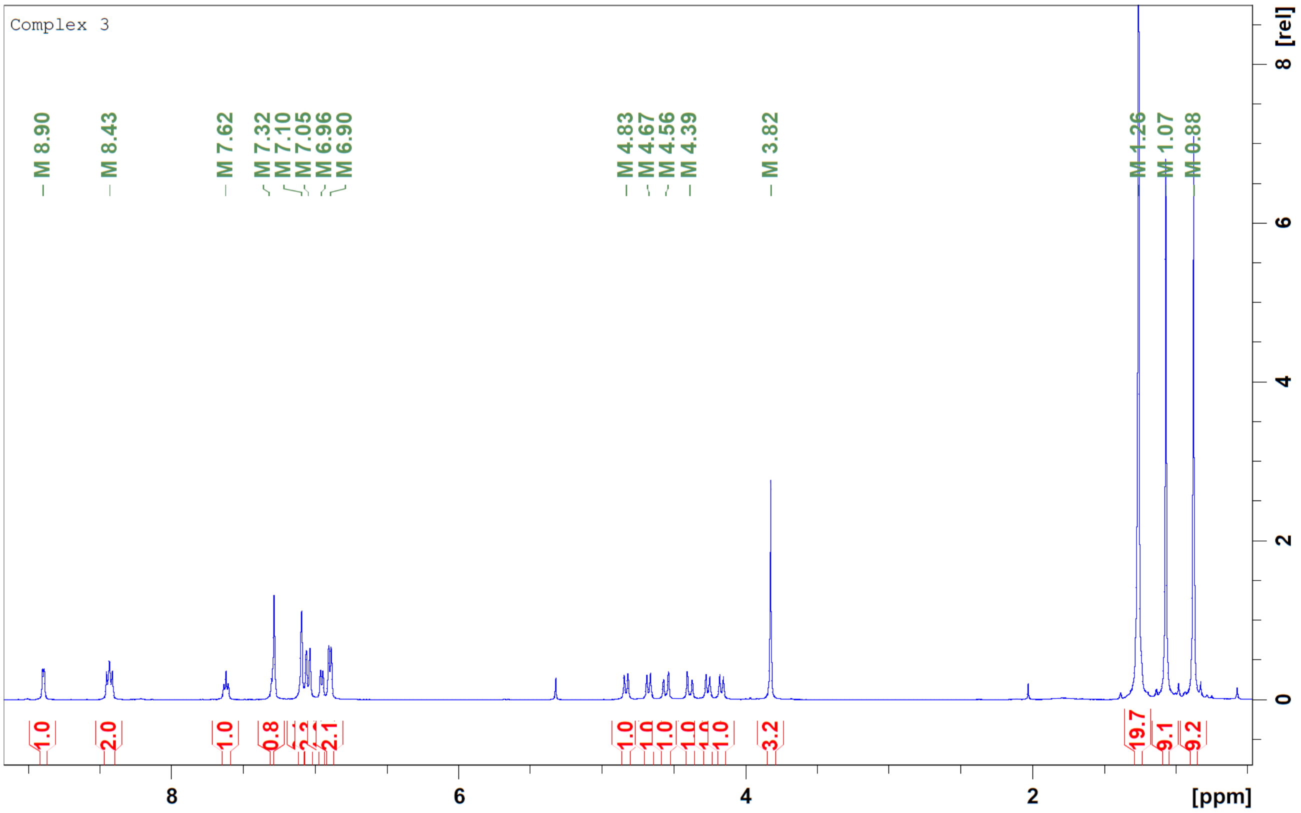

Figure S4. The ^{1}H spectrum of **3**. Peaks at 2.1 and 5.3 ppm are due to the acetonitrile of crystallization and CH_2Cl_2 residue used in the sample preparation, respectively.

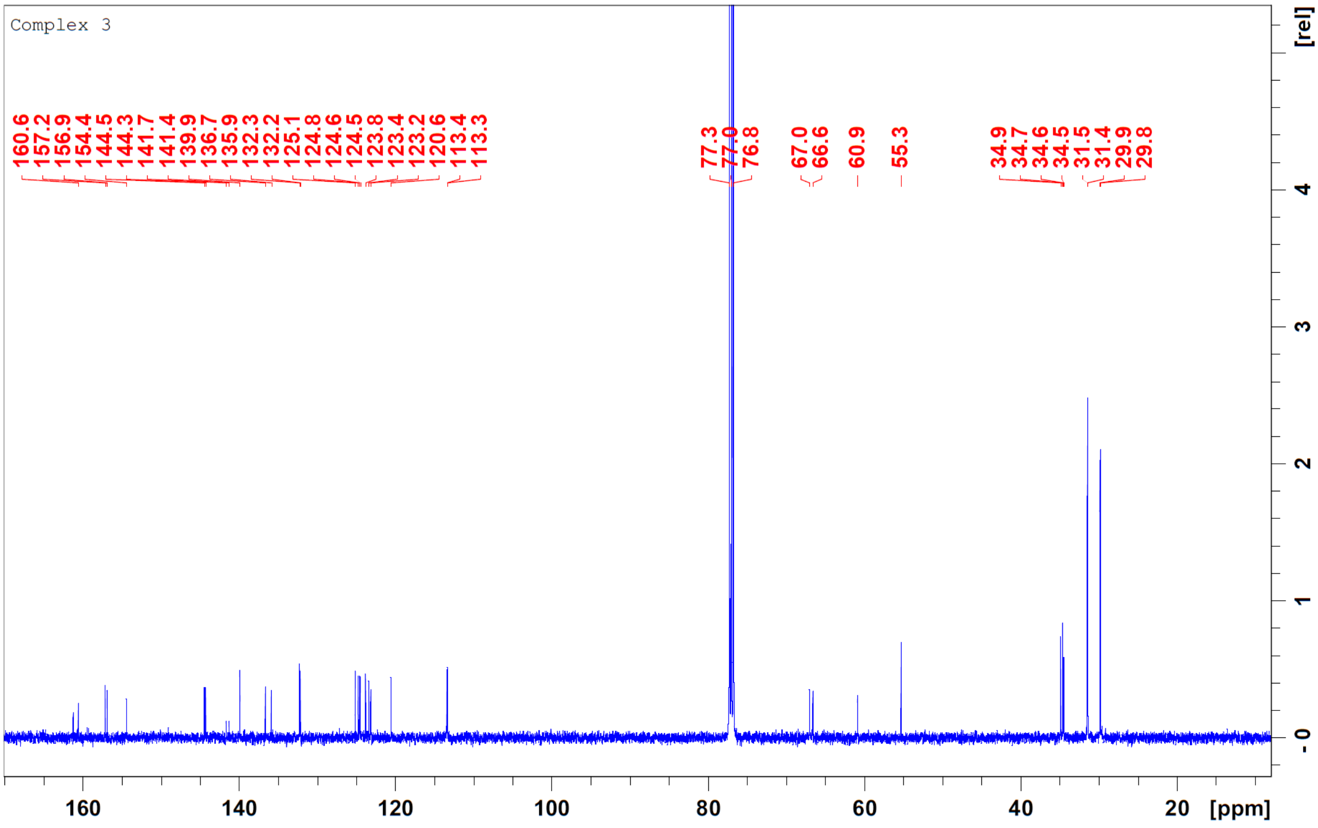

Figure S5. The ^{13}C NMR spectrum of **3**.
